# Fungal Traits Important for Soil Aggregation

**DOI:** 10.3389/fmicb.2019.02904

**Published:** 2020-01-09

**Authors:** Anika Lehmann, Weishuang Zheng, Masahiro Ryo, Katharina Soutschek, Julien Roy, Rebecca Rongstock, Stefanie Maaß, Matthias C. Rillig

**Affiliations:** ^1^Ecology of Plants, Institut für Biologie, Freie Universität Berlin, Berlin, Germany; ^2^Berlin-Brandenburg Institute of Advanced Biodiversity Research, Berlin, Germany; ^3^PKU-HKUST Shenzhen-Hong Kong Institution, Shenzhen, China; ^4^Plant Ecology and Nature Conservation, Institut für Biochemie und Biologie, Universität Potsdam, Potsdam, Germany

**Keywords:** soil aggregation, traits, saprobic fungi, random forest, biomass density, leucine amino peptidases

## Abstract

Soil structure, the complex arrangement of soil into aggregates and pore spaces, is a key feature of soils and soil biota. Among them, filamentous saprobic fungi have well-documented effects on soil aggregation. However, it is unclear what properties, or traits, determine the overall positive effect of fungi on soil aggregation. To achieve progress, it would be helpful to systematically investigate a broad suite of fungal species for their trait expression and the relation of these traits to soil aggregation. Here, we apply a trait-based approach to a set of 15 traits measured under standardized conditions on 31 fungal strains including Ascomycota, Basidiomycota, and Mucoromycota, all isolated from the same soil. We find large differences among these fungi in their ability to aggregate soil, including neutral to positive effects, and we document large differences in trait expression among strains. We identify biomass density, i.e., the density with which a mycelium grows (positive effects), leucine aminopeptidase activity (negative effects) and phylogeny as important factors explaining differences in soil aggregate formation (SAF) among fungal strains; importantly, growth rate was not among the important traits. Our results point to a typical suite of traits characterizing fungi that are good soil aggregators, and our findings illustrate the power of employing a trait-based approach to unravel biological mechanisms underpinning soil aggregation. Such an approach could now be extended also to other soil biota groups. In an applied context of restoration and agriculture, such trait information can inform management, for example to prioritize practices that favor the expression of more desirable fungal traits.

## Introduction

Soil is our most vital resource, with soil and its biodiversity contributing to many ecosystem processes (Bardgett and van der Putten, 2014), and to human nutrition, health and wellbeing (Wall et al., 2015). Soil has been described as the most complex biomaterial on Earth (Young and Crawford, 2004) with soil structure as one of its most important features. Soil structure represents the three-dimensional arrangement of soil particles into aggregates and associated pore spaces and is also a crucial parameter for sustainable management of soils (Bronick and Lal, 2005); therefore, it is of great interest to unravel how soil biota contribute to the process of soil aggregation.

Many soil biota influence soil aggregation (Lehmann et al., 2017b), and among them are the filamentous fungi. These fungi have a particularly well-documented impact on soil structure especially at the macroaggregate (>250 μm) scale, as highlighted in a meta-analysis (Lehmann et al., 2017b). Soil aggregating capability of fungi is hypothesized to be due to a range of physical, morphological, chemical and biotic traits (Six et al., 2004; Bronick and Lal, 2005; Lehmann et al., 2017a). While foraging and growing through soil, fungi are thought to entangle and enmesh soil particles and aggregates due to their filamentous growth form (Tisdall and Oades, 1982). Fungi also exude extracellular biopolymers which can act as cements and surface sealants for soil aggregates (Chenu, 1989; Caesar-TonThat and Cochran, 2000; Daynes et al., 2012), and enzymes degrading organic matter (Baldrian et al., 2011), which may serve as aggregate-disintegrating agents. Among the molecules they release are also hydrophobins, which can modify wettability of aggregates, likely serving a stabilizing function (Zheng et al., 2016). While growing through soil, fungi also interact with other members of the soil community, for example they can be grazed upon by Collembola, which can also influence soil aggregation ability (e.g., Siddiky et al., 2012a, b).

Fungi likely differ in many of these traits, and thus also in their soil aggregation capability. In fact, exploring a global dataset of fungal contributions to soil aggregation, Lehmann et al. (2017b) revealed a wide range in soil aggregation effectiveness for the 117 species for which experimental data were available. However, in this analysis it remained unclear which fungal traits underpin the observed effects on soil aggregation, simply because the relevant trait data are unavailable.

There is a need for studies that systematically compare fungal traits in a set of species and relate these traits to soil aggregation ability. So far, a relatively limited number of such studies are available (Supplementary Table S1), representing pioneering studies that have mainly focused on fungal biomass and some chemical traits, using specific fungal groups, such as arbuscular or ectomycorrhizal fungi. Much less is known for soil saprobic fungi. In all these studies, researchers have focused on a smaller set of fungi (typically in the range of 3–9 species), which were examined for some selected traits (no more than three traits). In cases where larger suites of fungi (up to 85 fungal strains/mutants) were investigated for their soil aggregation ability, no traits were measured, likely because of logistical limitations (Supplementary Table S1). Overall, we are thus not in a position where broadly generalizable conclusions can be drawn about fungal traits important for soil aggregation.

One approach to address this issue is by applying a trait-based approach, especially for saprobic fungi (Lehmann and Rillig, 2015). As opposed to arbuscular mycorrhizal fungi, for which most work in this context has been done (Rillig et al., 2015), there are also clear traits for disaggregation ability in saprobic fungi, which are related to enzymatic traits. In a trait-based approach, using a reasonably large suite of isolates, organismal traits can be related to specific functions. Such approaches generally convert species into points in “trait-space,” thus overcoming some limitations associated with examining a few selected strains, and thus allowing for more generalizable inferences (Crowther et al., 2014; Aguilar-Trigueros et al., 2015), at least within the confines of the set of fungi chosen for this purpose.

Here, we investigated a set of 31 filamentous fungal strains, all saprobic fungi isolated from the same soil, and thus representing one particular ecological context. We compared these strains under identical conditions in the laboratory; this is an important advantage compared to literature syntheses, since often isolate-specific growth media and conditions are used. The 31 strains are distributed among the Ascomycota, Basidiomycota and Mucoromycota (Spatafora et al., 2016), and we screened each for the expression of a suite of 15 traits. With these data, we wished to determine (i) which morphological, chemical and biotic traits are most important for soil aggregation and (ii) what characterizes an efficient or poor soil aggregator.

## Materials and Methods

### Soil and Fungal Strains

**FIGURE 1:**
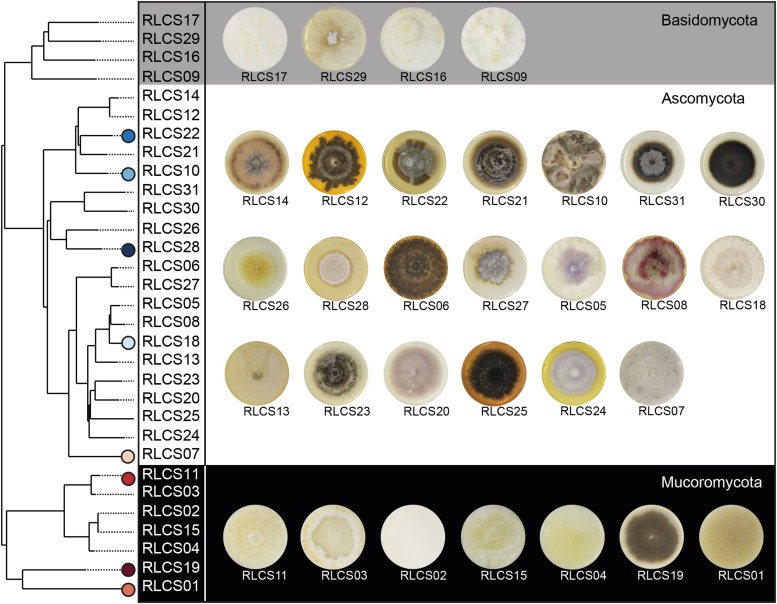
Overview of fungal strains. Phylogenetic tree (maximum clade-credibility tree) of the 31 saprobic fungal strains comprising members of the phyla Ascomycota, Basidiomycota, and Mucoromycota. Following the order of the tree, images of 4 week old colonies grown on PDA are assigned to the tree. Further information about phylogeny and accession numbers of the 31 fungal strains are available in Supplementary Table S2. Strains performing best and poorest are marked; blue symbols represent good and red symbols poor aggregators.

Soil samples and fungal strains were obtained from Mallnow Lebus, a dry grassland in a natural reserve (Brandenburg, Germany, 52° 27.778′ N, 14° 29.349′ E) characterized by a sandy loam soil texture. The collected soil samples were either used for establishing fungal cultures or were air-dried and stored until further use in experiments. The isolation of the 31 fungal strains was previously described in Andrade-Linares et al. (2016). Briefly, washed and diluted soil was used for the isolation procedure to minimize spore abundance and to increase the probability of capturing fungi derived from hyphae attached to soil particles (Gams and Domsch, 1967; Thorn et al., 1996). Afterward, soil suspensions were incubated on a variety of media with applications of different antibiotics (streptomycin, penicillin G, and chlortetracycline) suitable for cultivation of Ascomycota, Basidiomycota and Mucoromycota while suppressing bacterial growth. Isolates were grown on PDA at room temperature (22°C). Our final set of fungal strains comprised 20 Ascomycota, four Basidiomycota, and seven Mucoromycota strains (Figure 1 and Supplementary Table S2).

The inference of the phylogenetic relationships of the 31 fungal strains was based on the complete intergenic transcribed spacer (ITS) and a part of the large rRNA subunit (LSU) (Lehmann et al., 2019b). Fungal DNA was extracted using Qiagen DNeasy PowerSoil Kit (100) or MasterPure Yeast DNA Purification Kit (Epicenter, Madison, WI, United States) while following manufacturer’s instructions. We amplified the ITS and partial LSU regions via ITS1F and LR5 primers. The internal primers ITS4 and NL4 were used for sequencing with Sanger technology. We applied the software tool ITSx (Bengtsson-Palme et al., 2013) to split the rRNA sequences into the subregions ITS1, ITS2, LSU, and 5.8S. Each region was aligned independently using AlignSeqs in the R package DECIPHER 2.0 (Wright, 2015, 2016). Subsequently, the aligned subregion sequences were concatenated and pairwise distances calculated via JC69 evolutionary model. We then constructed a neighbor-joining tree (Figure 1) by applying the dist.ml() and NJ() functions, respectively, of the R package “phangorn” 2.5.5 (Schliep, 2011). The root was placed at the midpoint of the longest path between any two tips. Finally, we inferred the taxonomic annotations of the fungal isolates based on each subregions by appliying the Naive Bayesian Classifier (Wang et al., 2007) implemented in the R package “dada2” (Callahan et al., 2016). For ITS1 or ITS2 sequences, we investigate the UNITE database (Nilsson et al., 2019), while for LSU sequences we used the RDP LSU database (Cole et al., 2011). We verified the annotations following a confidence threshold approach incorporating bootstrap analysis; an annotation was valid if it was supported in 80% of the bootstraps. We chose the best-resolved taxonomic annotation among the investigated regions. In case of conflicting taxonomic annotations for the different investigated regions, we gave priority to ITS1 or ITS2 over LSU since the UNITE database is more complete than the RDP LSU database. We followed the phylum classification by Spatafora et al. (2017) (Supplementary Table S1).

### Soil Aggregate Formation

The soil aggregate formation (SAF) assay used here aimed to test for *de novo* aggregate formation by fungi. This technique was modified from Tisdall et al. (2012). Here, we filled 6 cm petri dishes with a 5 mm layer of agar (1.5%, Panreac AppliChem, Darmstadt, Germany) to provide moisture, and this layer was covered with 10.0 g of soil. The soil was gently poured onto the agar to avoid any artificial compaction. Prior to this, the soil (from the field site from which the fungi were originally isolated) was sieved to a fraction <1 mm and autoclaved two times in a dry cycle. The soil was then allowed to equilibrate for 2 days on the agar before inoculation. During this time, the soil was rewetted by capillary action. This way, we provided a moist but not waterlogged environment for the fungal strains. The fungal strains used for inoculation were cultured on PDA and incubated with sterilized poppy (*Papaver somniferum*) seeds as carrier material. Colonized poppy seeds were transferred to soil – with two seeds per species added per soil plate. For the controls, non-colonized poppy seeds incubated on PDA were transferred to the soil plates. Finally, plates were sealed and stored at room temperature (22°C, the culturing temperature of our fungal strains) in the dark for 6 weeks until harvest. The experiment consisted of ten replicates for 31 fungal strains and a control, resulting in 320 experimental units.

We visually confirmed for every strain (on two replicates) that hyphae were not just growing on the surface of the soil, but that the mycelium was present inside the soil. At harvest, the plates were opened and dried at 60°C overnight. Subsequently, the soil was carefully extracted from the Petri dishes, passed through a 1 mm sieve to extract all aggregates larger than 1 mm, which were formed during the experiment. To do so, we vertically moved the sieve two times to allow separation while avoiding abrasion of soil aggregates. Additionally, we tapped against the sieve frame. By this, we increased the likelihood of passing aggregates and particles <1 mm captured by hyphae through the mesh. The weight of the soil fraction >1 mm was used for the calculation of the SAF for our 31 fungal strains and the corresponding controls following the equation: % SAF = (aggregates_> 1 mm_/10.0) × 100.

This approach offers the opportunity to test SAF for an *a priori* size fraction (here 1 mm). However, this design does not capture any dynamics for the <1 mm soil fraction. Hence any impact of the 31 fungal strains on e.g., microaggregate formation could not be evaluated here.

### Trait Measurements

To build a trait database, we investigated 15 different traits capturing morphological, chemical and biotic features of our 31 fungal strains (Lehmann and Rillig, 2015; Lehmann et al., 2017a). The traits were chosen to characterize different aspects of the fungal mycelium and its products by which the fungus interacts with its environment. Additionally, the traits had to be measurable for all 31 strains, using methods that worked for all of them. The trait data were either obtained from dedicated new experiments or collected from previously published studies (Zheng et al., 2018; Lehmann et al., 2019b) using the set of 31 fungal strains; data origin is given in the text.

With the exception of hyphal length, all traits were measured under standardized *in vitro* conditions which were suitable for all our fungal strains. It was not feasible to realize trait measurements in soil since it is an opaque and highly heterogeneous substrate. Instead we used potato dextrose agar, a widely used standard growth medium for fungi. By this, we ensure a consistent environmental setting for trait measurements (Aguilar-Trigueros et al., 2015; Lehmann and Rillig, 2015).

#### Morphological Traits

We measured hyphal length in soil (in m g^–1^ soil); for this we used soil samples from the SAF assay; hence we had ten replicates for each fungal strain and the control. For extracting hyphae and measuring hyphal length, 4.0 g of the experimental soil were used, and hyphae counted at 200× magnification (Tennant, 1975; Jakobsen et al., 1992). The hyphal length found in the controls was set as the background; that is, dead hyphae that were present in the soil after autoclaving.

In order to measure colony radial growth rate (in μm h^–1^), the 31 fungal strains were cultivated on full strength PDA – a rich medium generally preventing growth limitations in our fungal strains. For each fungal strain five replicates were used. For the set-up, a pre-sterilized poppy seed colonized by a fungal strain was placed in the center of a PDA plate which was then incubated for 4 weeks in the dark at room temperature (22°C). At day 0, 3, 5, 7, 14, 21, and 28, all plates were scanned from the back with an Epson Perfection V700 Photo Scanner (300 dpi, 16-bit, color). The pictures were analyzed in ImageJ (Schneider et al., 2012) (1.51j8) by measuring the radius in four directions (0°, 90°, 180°, and 270°) with the poppy seed as center point to the colony rim. The four values were averaged. For each replicate, the mean colony radius was plotted over time to identify the linear growth phase. The slope of the linear growth phase represents the colony radial growth rate and was estimated by linear regression standardized by the length of the linear growth phase.

The data for colony biomass density (in μg mm^–2^) were obtained in an experiment in which fungal colonies were grown on PDA covered with sterilized cellophane, allowing easy extraction of fungal biomass. For each fungal strain, six replicates were set up using colonized poppy seeds, as above. When fungi reached half of their linear growth phase, colony area was measured, then biomass was harvested, dried at 45°C and weighed. Finally, the biomass was standardized by the colony area (Reeslev and Kjoller, 1995).

Furthermore, we included data on hyphal branching angle (BA), hyphal internodal length (IL), hyphal diameter, mycelial complexity (box counting dimension, describing the degree of detail of a pattern), and mycelium heterogeneity [lacunarity, i.e., the gappiness or “rotational and translational invariance” in a pattern (Karperien, 1999-2013)] and hyphal surface area (HSA) which were collected by Lehmann et al. (2019b). For further information on experimental set-up and measurements see Supplementary Material.

#### Chemical Traits

We measured hydrophobicity of the fungal surface for fungal material using the same approach as applied for biomass density measurements, with six replicates per fungal strain. This allowed us to use medium-free fungal material. Half of an individual colony was used for the hydrophobicity test, which was done using alcohol percentage tests. This is a rapid and simple way of quantifying hydrophobicity (Chau et al., 2010). Briefly, a series of ethanol droplets (8 μl) with a concentration gradient were placed on the fungal surface to find the maximum concentration at which the droplet can retain its shape for longer than 5 s (Zheng et al., 2014).

Additionally, we included here the enzymatic activity data for laccase, cellobiohydrolase, leucine aminopeptidase and acid phosphatase, previously measured by Zheng et al. (2018). For further information, see Supplementary Material.

#### Biological Trait

The palatability of the 31 fungal strains was tested in a feeding experiment with the collembolan *Folsomia candida*. We measured palatability as a proxy for assessing likely persistence of hyphae in the environment, as a way to assess possible interaction with other soil biota. Fungal mycelium was grown on glass fiber filter papers (696, VWR European Cat. No. 516-0877) cut into 1 cm^2^ pieces of which four were placed in Petri dishes filled with plaster of Paris and charcoal (3:1 mixture). There were 31 fungal treatments and a non-fungal control (glass fiber filters only), each with eight replicates resulting in 256 experimental units. The experiment started with the addition of ten individuals of Collembola of the same age and developmental stage; the animals were previously starved for 7 days. After 3 days of incubation in the dark at room temperature (22°C), experimental units were checked for numbers of alive Collembola and subsequently were frozen at −20°C to stop any activity. Finally, the number of fecal pellets per dish were measured and standardized by number of surviving Collembola (fecal pellets × no. of individuals^–1^).

### Statistics

First, for investigating SAF capability of the 31 fungal strains, we tested fungal performances against the corresponding control samples using a generalized least square model (gls with *n* = 10 × 32 = 320) in the “nlme” package (Pinheiro et al., 2018); we accounted for heteroscedasticity by implementing different variances per stratum for fungal strains by using the varIdent function (Zuur et al., 2009). To test for differences in SAF performance of different phyla we used analysis of variance (*n* = 31) with subsequent pairwise comparisons via TukeyHSD() function. For all models, we tested for normality and homogeneity of model residuals.

Second, we applied principal components analysis to investigate the 15-dimensional trait space and the distribution of fungal strains therein. For this, we used the prcomp() function in the basic “stats” package; we used *z*-transformed data. To reduce the dimensionality of our dataset we tested for PC axis significance via the function testdim() (Dray, 2008) in the package “ade4” (Chessel et al., 2004; Dray and Dufour, 2007; Dray et al., 2007). We found that the first two axes were significant and hence used these for the PCA biplot. We added species occurrence probability information to the biplot by applying the kernel density estimation following the approach of Diaz et al. (Diaz et al., 2016). For this, we used the kde() function in the package “ks” (Duong, 2018) and implemented an unconstrained bandwidth selector via the function Hpi() for our first two PC axes. We estimated contour probabilities for 0.5, 0.95, and 0.99 quantiles with the function contourLevels(). Additionally, we tested for collinearity between our 15 trait variables by using Pearson’s rho. A threshold of | rho| >0.7 was defined as an indicator of collinearity (Dormann et al., 2013).

Third, we applied a permutation-based random forest algorithm (Hapfelmeier and Ulm, 2013) to identify informative trait variables which are important for SAF. Random forest (Breiman, 2001) is one of the machine learning algorithms with highest accuracy (Douglas et al., 2011; Crisci et al., 2012), and is capable of detecting non-linear relationships even among higher order interactions in a non-parametric manner (Ryo and Rillig, 2017; Ryo et al., 2018), while being robust to multicollinearity (Nicodemus et al., 2010). SAF was regressed with all the trait variables, and the model performance was evaluated in terms of explanatory power (i.e., variability explained, *R*^2^_*expl*_) and predictability using out-of-bag cross validation (Breiman, 1996) (*R*^2^_*pred*_). The relative importance of the trait variables was quantified with a mean squared error measure, indicating how much each of the trait variables contributes to the model predictability (Breiman, 2001). In addition, statistical significance of each trait variable (*p* = 0.05) was tested via a permutation approach with 2000 iterations (Hapfelmeier and Ulm, 2013). The two parameters of the random forest algorithm (see Breiman, 2001) were tuned as follows: the number of trees in the model (ntree) was set to 100 as it made the model stable (Breiman, 2001); the number of predictors for the randomized split technique (mtry) was set to 4 [the square root of the number of predictors (Diaz-Uriarte and de Andres, 2006)].

We added the phylogeny of our 31 fungal strains as a numeric predictor variable to the random forest analysis. To do this, we calculated phylogenetic pairwise distances and fed these into PCoA via the cmdscale() function in the “stats” package. We calculated the cumulative sum of the proportion of variance explained by PCo axes based on the eigenvalues and extracted the first five axes, together explaining up to 80% of phylogenetic variance (Diniz-Filho et al., 1998). The PCo axes were integrated as five individual variables in the random forest analysis. After identifying the most relevant predictors, we used partial dependence plots to visualize the response-predictor relationships obtained from the random forest procedure (Hastie et al., 2009). For this, we used the plotPartialDependence() function of the package “mlr” (Bischl et al., 2016).

Fourth, we tested for phylogenetic signals in our 15 trait variables (Supplementary Table S3) using Moran’s I statistic – a measure of phylogenetic autocorrelation, implemented in the package “phylosignal” (Keck et al., 2016).

Fifth, we ran linear regressions on SAF and the three most important predictors identified by the random forest approach and further evaluated the relationships by quantile regression with the package “quantreg”^1^. Analyzing response–predictor relationships at their maxima rather than at their means allows for more meaningful inferences especially for wedge-shaped data distributions (Cade et al., 1999; Cade and Noon, 2003); in these cases, unmeasured limiting factors could obscure underlying patterns. Model residuals were tested for homogeneity and normal distribution. If necessary, data were log-transformed.

Sixth, we visually explored SAF strategies exemplified by the four best and poorest performing strains via radar charts applying the eponymous function in the package “fmsb” (Nakazawa, 2018).

We conducted all analyses in R (R Development Core Team, 2014) (v. 3.4.1) and generated plots, if not stated otherwise, with the graphic package “ggplot2” (Wickham, 2009).

## Results and Discussion

### Soil Aggregate Formation

**FIGURE 2:**
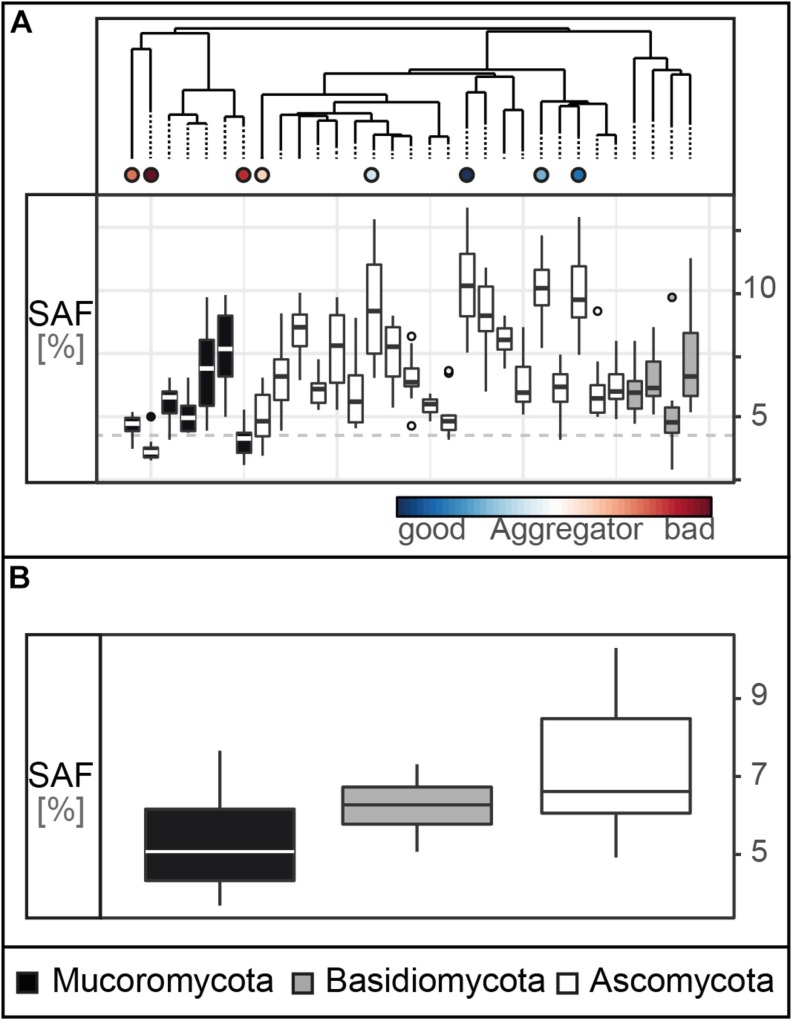
Soil aggregate formation (SAF) capability. **(A)** Tukey boxplots of the SAF (with *n* = 10 × 31 in %) capability of the 31 fungal strains. The dashed line represents the average SAF of the controls (*n* = 10, mean = 3.5, SD = 0.59). **(B)** SAF capability depicted on phylum level (pairwise comparisons: Ascomycota – Basidiomycota: *p* = 0.47, Ascomycota – Mucoromycota: *p* = 0.03, Basidiomycota – Mucoromycota: *p* = 0.66; *n* = 31).

We here measured SAF capability on a broad set of fungal strains comprising the phyla Ascomycota, Basidiomycota and Mucoromycota, revealing an overall significantly positive effect of fungi on soil aggregation: the saprobic fungi increased SAF of the tested sandy soil by 79% (confidence interval: 61–99%; Supplementary Figure S1) compared to the non-inoculated controls. The control samples reached a SAF of 3.5% (standard deviation: 0.6) while, for the fungal treatments, we found a spectrum of SAF with means ranging from 3.7 to 10.3% with the Mucoromycota strain RCLS19 and the Ascomycota strain RLCS28 at the lower and upper end, respectively (Figure 2A). Only two strains, namely RCLS19 and RCLS11, had a SAF performance not significantly different from the non-inoculated controls.

Our results support the general finding that filamentous soil fungi improve soil aggregation, as was shown in experiments (Martin and Anderson, 1943; Gilmour et al., 1948; Martin et al., 1958; Zheng et al., 2014) and a global data synthesis (Lehmann et al., 2017b). However, here we used for the first time a set of 31 fungal strains comprising three major fungal phyla which were all isolated from the same soil and tested in their home soil. This set was screened using a method suitable for the large number of target species. Additionally, we used a straightforward assay for testing specifically a soil aggregation process component – namely aggregate formation.

Our choice of methods also has limitations. Using this approach, we only focused on one *a priori* size limit for newly formed aggregates, thus not capturing any dynamics in smaller sizes classes. Furthermore, the small amount of soil used in our design did not allow us to measure aggregate size distributions. We here evaluated fungal contributions to soil aggregation in isolation, not taking into account how such effects might be modified by other soil organisms. However, such species interactions can be clearly important; for example, a recent meta-analysis revealed that soil biota combinations (e.g., bacteria–fungi mixtures) result in significantly increased soil aggregation (Lehmann et al., 2017b). Hence, future studies should also consider species combinations when evaluating soil biota contributions to soil aggregation.

In our experiment, each of the three tested fungal phyla contained strains that were effective and poorly performing; however, overall, the four most efficient aggregate formers were members of the Ascomycota while three of the poorest aggregate formers belonged to the Mucoromycota (Figure 2B). For our tested suite of fungi, we found that the Ascomycota, in general, had significantly higher SAF than the Mucoromycota. These findings correspond with previous reports (Lynch and Elliott, 1983; Tisdall et al., 2012; Lehmann et al., 2017b) and suggest that phylogeny is a strong factor determining SAF capability. However, it still remains unclear which fungal traits contribute to these phylum-specific differences and overall variability in SAF capability. Thus, in the next step, we used a trait database comprising morphological, chemical and biotic traits to explore their importance for SAF.

### Trait Collection

**FIGURE 3:**
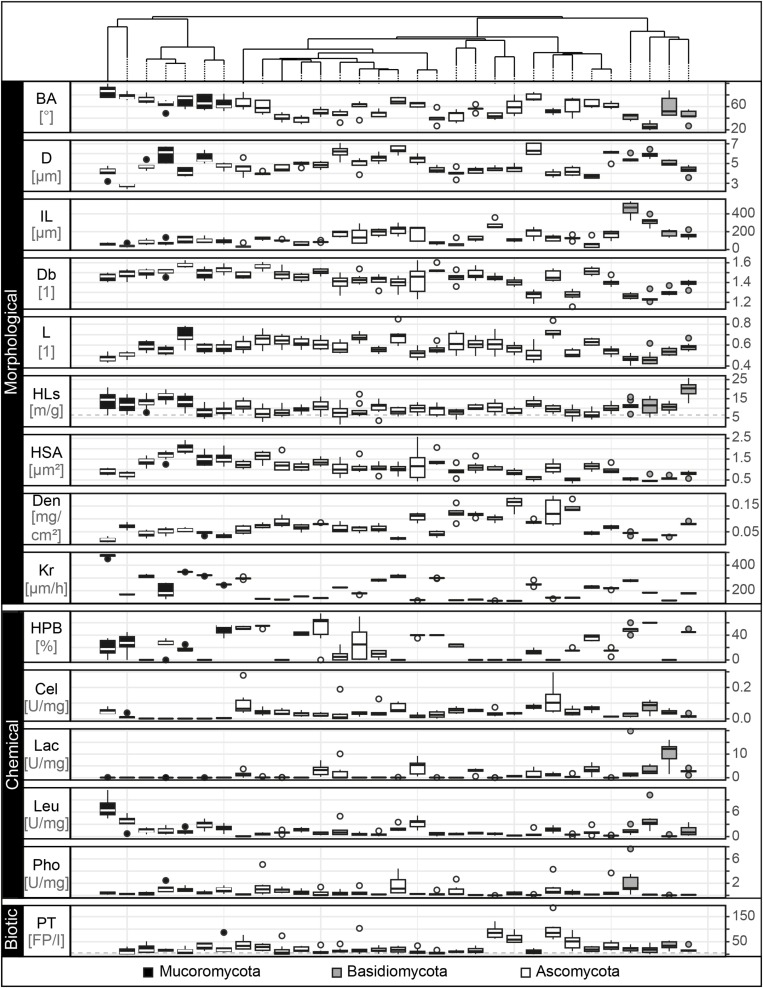
Trait distributions. Tukey boxplots of the 15 trait variables comprising morphological, chemical and biotic fungal features. Here, we present data on branching angle (BA with *n* = 5 in °), hyphal diameter (D with *n* = 5 in μm), internodal length (IL with *n* = 5 in μm), box counting dimension (Db with *n* = 8, unitless), lacunarity (L with *n* = 8, unitless), hyphal length in soil (HLs with *n* = 10 in m/g), hyphal surface area (HSA with *n* = 8 in μm^2^), biomass density (Den with *n* = 6 in mg × cm^– 2^), radial colony extension rate (Kr with *n* = 5 in μm × h^– 1^), hydrophobicity of fungal surfaces (HPB with *n* = 6 in% of ethanol molarity), cellobiohydrolase (Cel), laccase (Lac), leucine aminopeptidase (Leu), and acid phosphatase (Pho) activity (each with *n* = 5 in unit × g^– 1^ dry mass) and palatability (PT with *n* = 8 in no. of fecal pellets per collembolan individual). The boxplots represent 25th and 75th percentile, median and outlying points. Information about phylum affiliation is color-coded (black: Mucoromycota, gray: Basidiomycota, white: Ascomycota). The gray dashed line for the trait hyphal length in soil represents mean of corresponding trait controls. The trait database is available in Supplementary Table S6.

We included 15 fungal traits (measured on the level of a fungal individual or “colony”) and found strong variability in their expression across the 31 fungal strains (Figure 3). In terms of morphological features, we found in our experiments that the measured branching angles ranged from 26° to 86°, with Mucoromycota having the widest and Basidiomycota the narrowest angles, while for hyphal diameter, the highest and lowest values (2.7–6.5 μm) were both found in the Mucoromycota. Basidiomycota had the highest internodal length (453 μm) while in Mucoromycota distances as short as 40 μm between two branches were detected. The mycelium complexity measurements revealed trait values between 1.2 (Basidiomycota) and 1.6 (Mucoromycota), where a value of 1 represents a single, unbranched hypha and a value of 2 a complex, space-filling structure. Mycelium heterogeneity varied between 0.4 and 0.7 for Basidio- and Ascomycota, respectively, with higher values indicating increasing structural gappiness. For hyphal length in soil, we found 7 – 20 m hyphae per g soil for Ascomycota and Basidiomycota, respectively, with 4.6 m g^–1^ of hyphal background. The largest hyphal surface area was found in Mucoromycota with 3.4 μm^2^ while the smallest was detected for an Ascomycota strain with 0.8 μm^2^. For biomass density, values ranged between 0.02 and 0.2 mg cm^–2^ for Basidiomycota and Ascomycota, respectively. Among the Mucoromycota the strain with the highest colony radial extension rate with 373 μm h^–1^ was found while the slowest extending strain was a member of the Ascomycota.

Next, the exploration of the chemical traits revealed that hydrophilic mycelia could be found across all phyla, while Basidiomycota showed the strongest detectable mycelial hydrophobicity (60% ethanol molarity). The enzyme profiling revealed that cellobiohydrolase was not produced by Mortierellales, an order of the Mucoromycota, while the highest activity was found in the Ascomycota (0.13 U mg^–^1). In contrast, laccase and acid phosphatase activities were lowest in Ascomycota and highest in Basidiomycota (laccase: 0.01–10.4 U mg^–1^; acid phosphatase: 0.01–1.8 U mg^–1^). The production of leucine aminopeptidase was highest in Mucoromycota and lowest in Ascomycota (0.09–7.1 U mg^–1^).

We measured palatability as a biotic trait and found that the most and least attractive strains belonged to the Ascomycota (5–123 fecal pellets per individual collembolan).

Here, we established a collection of soft traits measured under standardized conditions with reproducible methods that are applicable for a broad range of fungal strains with high intra- and interspecific variability in morphological, chemical and biotic features. Our values are within the range of previously reported fungal traits (e.g., Trinci, 1969; Ho, 1978; Obert et al., 1990; Baldrian et al., 2011; Eichlerova et al., 2015).

However, it is important to note that these findings result from trait data measured on a homogenous, standardized growth substrate not accounting for the heterogeneous nature of soil with its inherent structure and also physical, chemical and biotic factors influencing the fungal trait expression. It is well known that fungal mycelia are versatile, dynamic and modular constructs; they not only modify their environment during foraging but also react to it (Ritz and Young, 2004). As demonstrated using the model organism *Rhizoctonia solani*, nutrient distribution and soil bulk density can alter e.g., hyphal growth patterns and thus mycelium density (Harris et al., 2003; Boswell et al., 2007). Future studies would need to take into account the soil heterogeneity.

### Fungal Trait Space

**FIGURE 4:**
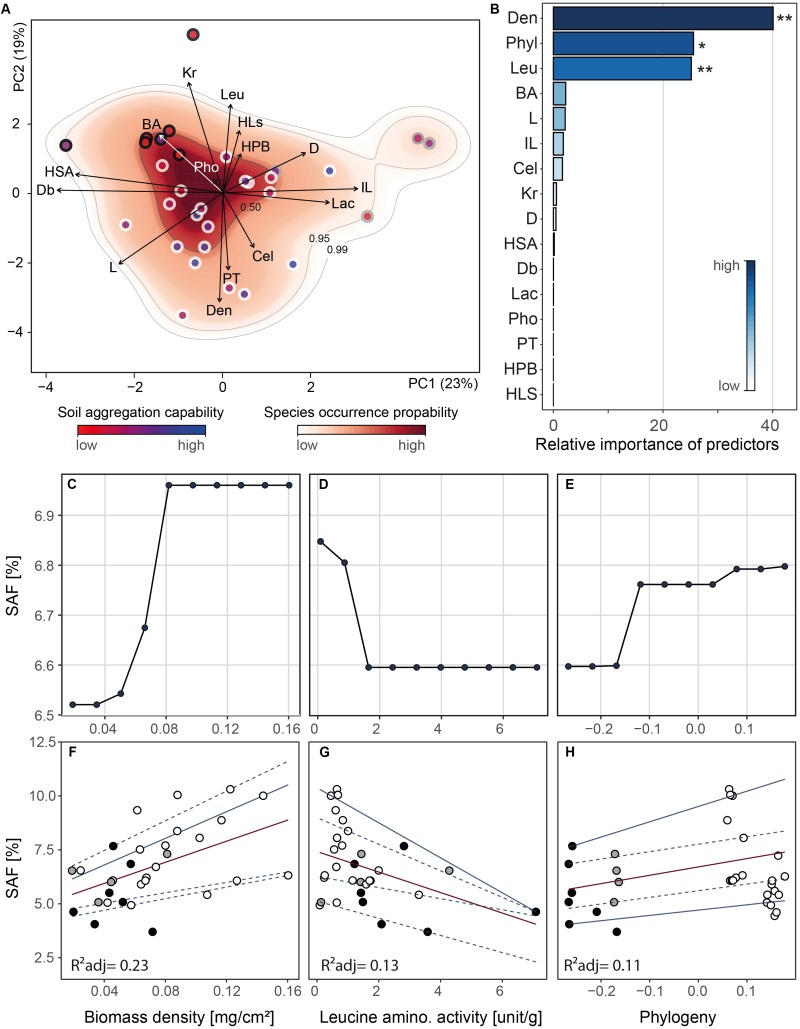
Outcomes of principal components analysis, random forest analysis and relationships between soil aggregate formation (SAF) and important trait variables. Analyses were conducted on trait mean data (*n* = 31). **(A)** Projection of the ordinated 31 fungal strains onto 15 trait variables comprising morphological, chemical and biotic characteristics into two dimensional trait space represented by principal component axis 1 and 2 (explaining 23 and 19% of variance, respectively). The trait variables are branching angle (BA), hyphal diameter (D), internodal length (IL), box counting dimension (Db), lacunarity (L), hyphal length in soil (HLs), hyphal surface area (HSA), biomass density (Den), radial colony extension rate (Kr), hydrophobicity of fungal surfaces (HPB), cellobiohydrolase (Cel), laccase (Lac), leucine aminopeptidase (Leu) and acid phosphatase (Pho) activity, and palatability (PT). Arrows indicate direction and weight of trait vectors. Red–white color gradient represents probability of species occurrence (white = low, red = high) in the trait space, with the contour lines denoting the 0.50, 0.95, and 0.99 quantiles of kernel density estimation (see “Materials and Methods” section). The dot outline represents phylum affiliation (black: Mucoromycota, gray: Basidiomycota, white: Ascomycota) while dot filling represents soil aggregate formation capability (SAF) of fungal strains (represented by a blue–red color gradient; red: low SAF, blue: high SAF). **(B)** Overall importance of trait variables for SAF capability with *R*^2^_*expl*_ = 0.36, 0.13 and three statistically significant predictor variables. Asterisks denote significance level: ^∗∗^<0.001, ^∗^<0.01, <0.5. Pairwise phylogenetic distance was included as PCo-axes (see “Materials and Methods” section). **(C–E)** Partial dependence plots for the three most important and significant trait variables identified by random forest approach. For phylogeny, we depicted PCo axis 1 on the *x*-axis representing the axis scores. The *x*-axis labels are identical with panels **(F–H)**, respectively. **(F–H)** Relationships between SAF and the three most important trait variables. Corresponding regression statistics can be found in Supplementary Table S5. Phylum affiliation of fungal strains is color-coded (black: Mucoromycota, gray: Basidiomycota, white: Ascomycota). Red and blue lines represent linear and quantile regression lines, respectively. The line type depicts significance of regression lines with solid <0.05 and dashed >0.05. The trait database is available in Supplementary Table S6.

We investigated the resulting 15-dimensional trait space and the fungal strain probability occurrence therein (Figure 4A). We constructed the trait space by ordination (principal components analysis) and hence converted individual strains into unique trait combinations whose coordinates are determined by their trait expression (Crowther et al., 2014; Aguilar-Trigueros et al., 2015). We found that 42% of the variability in the fungal traits was accounted for in the first two PC axes which were the only significant axes (Supplementary Table S4). Due to indication of strong trait correlations, we tested our data for collinearity. We detected only one case of collinearity (| Pearson’s rho| > 0.7) for mycelium complexity and hyphal surface area (Supplementary Figure S2).

Evaluating the species occurrence, we found that Ascomycota strains were distributed in the lower half of the PC plane whereas the Mucoromycota were localized in the upper left quadrant mainly characterized by hyphal branching angle, colony radial growth rate and leucine aminopeptidase activity. In the upper right quadrant, the Basidiomycota grouped driven by hyphal internodial length and lacunarity. There was a clear separation of the phyla detectable for PC axis 1 with Ascomycota flanked by Mucoromycota and Basidiomycota but only a marginal separation between Ascomycota and Mucoromycota on PC axis 2 (Supplementary Figure S3). In general, the trait space revealed a high versatility in our fungal set with no clear syndromes. However, on the phylum level a clear separation between the three phyla was evident (Supplementary Figure S3).

In the next step, we investigated the importance of the collected fungal traits on SAF using the random forest approach. Considering the strong impact of phylum on SAF and phylogenetic separation in the trait space, we included phylogenetic pairwise distances as an additional variable (potentially also capturing not explicitly measured variables) in the following analyses.

### Fungal Trait Contributions to Soil Aggregate Formation

The random forest algorithm (explanatory power: 36% and predictability: 13%), identified three significant trait variables: colony biomass density, leucine aminopeptidase activity and phylogeny (relative importance: 48, 25, and 13%, explanatory power of each: 17.3, 9, and 4.7%; Figure 4B). Among the five phylogeny-encoding PCo axes only for axis one a relevance for SAF could be detected.

To visualize the modeled relationship between SAF and the important variables we used partial dependence plots. After taking into account the effects of all predictors except for the variable of interest (colony biomass density, leucine aminopeptidase activity or phylogeny, respectively), partial dependence plots depict the relationships between the predictor and the response variable (SAF). We found that SAF increased with increasing colony biomass density (Figure 4C) but decreased with increasing leucine aminopeptidase activity (Figure 4D). Across the phylogeny, from Mucoromycota to Ascomycota, we found a positive relationship with SAF (presenting phylogeny PCo axis 1, Figure 4E). These findings were supported by linear and quantile regression analyses (Figures 4F–H and Supplementary Table S5). Here, we found that the relationship between SAF and colony biomass density was best represented by mean regression. For the relationships between SAF and leucine aminopeptidase activity as well as SAF and phylogeny, the 0.95 and 0.05 quantile, respectively, showed the highest fit.

Our analyses revealed that fungal strains belonging to the Ascomycota that have high biomass density and low leucine aminopeptidase activity have the highest probability to form aggregates compared to other strains. Furthermore, we found that a colony biomass density above 0.08 mg cm^–2^ and a leucine aminopeptidase activity less than 1.8 U g^–1^ do not further improve SAF (Figures 4C,D).

Our findings further support the assumption that phylogeny influences aggregate forming capability of fungi (Figures 2B, 4H). We interpret this to mean that traits (including unmeasured traits) expressed by strains of the Ascomycota contribute to this beneficial impact on soil aggregation. Considering all possible traits and their expression, the four most efficient aggregate former were all Ascomycota with low leucine aminopeptidase activity and dense mycelia.

A densely growing fungus likely can more intensively cross-link and enmesh particles with its hyphae, and thus perhaps is more effective at contributing to the formation of macroaggregates; however, so far there has not been direct evidence of this. Interestingly, the total amount of hyphae produced was not an important explanatory variable (Figure 2; HLs = hyphal length in soil) suggesting that a critical local density is much more important than total hyphal production. This also explains results from previous experiments, where total hyphal length or biomass did not predict soil aggregation effects (e.g., Piotrowski et al., 2004). Fungi with high biomass density had low radial colony extension rate (Supplementary Figure S2); thus it can be expected that their positive effect on SAF is highly localized not reaching beyond their area of mycelial influence.

Fungi with low leucine aminopeptidase activity are inefficient in hydrolyzing peptides and thus degrading organic matter components, which may be functioning as glues and cementing agents in aggregates (Chenu, 1989; Caesar-TonThat and Cochran, 2000; Daynes et al., 2012). Fungi with either one of these traits are more likely able to bring soil particles and aggregates together via their hyphae; lacking the enzyme to degrade organic matter holding together aggregates also contributes to this effect.

**FIGURE 5:**
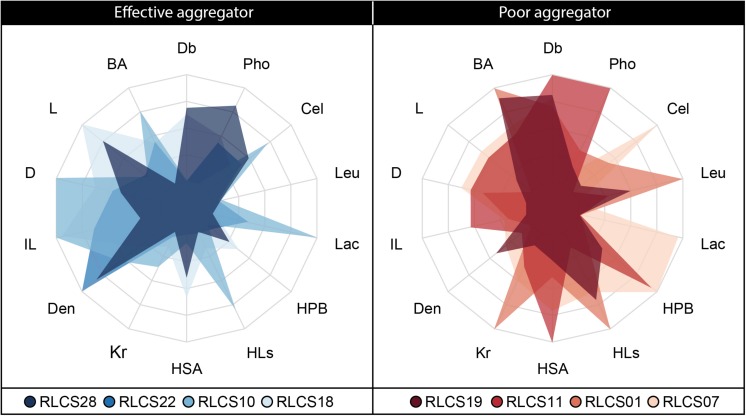
Radar plot depicting trait expressions for the four best and four poorest soil aggregate forming fungal strains.

After identifying the most important fungal traits for SAF, we focused on those fungi that are present at the lower and upper end of the SAF spectrum. The most efficient strains were all members of the Ascomycota (RLCS28, RLCS22, RLCS10, RLCS18) while the group of the poor performer comprised mainly Mucoromycota but also one ascomycete (RLCS19, RLCS11, RLCS01, RLCS07) (Figures 1, 2). As expected, the efficient soil aggregate forming strains had high biomass density but low leucine aminopeptidase activity (Figure 5). The opposite was true for the poor performers. In addition to these two clear features, the efficient strains tended to have lower colony radial growth rates, hyphal surface area and surface hydrophobicity, but had larger hyphal diameters and more heterogeneously structured mycelia as the four poorest soil aggregators.

The interpretation of our data is limited to our set of 31 fungal strains which is dominated by Ascomycota isolates. Additionally, we here used test systems with defined environmental conditions including a soil with high sand content. In such soils, fungi are an essential factor in soil aggregation mainly via physical and chemical interactions of hyphae with sand particles forming and stabilizing the otherwise unstable substrate (Sutton and Sheppard, 1976; Forster and Nicolson, 1981). We here chose the soil from which our fungi were originally cultured. For future studies, it would be interesting to extend our research to other sets of fungi under varying environmental conditions. Such an effort would improve the external validity and offer new insights into the mechanisms of fungal trait contributions to soil aggregation.

## Conclusion

Our results yield new insights into fungal traits important for soil aggregation, and thus also shed light on mechanisms of soil aggregation. Clearly, future work should focus on hyphal density as a key trait. In an applied context of restoration and agriculture, our trait information can be incorporated in management practices affecting the fungal environment in soil to favor the development of more dense fungal mycelia by e.g., carbon input or through a screen for isolates exhibiting desired traits under the soil conditions in which they will be used.

Even though we here focused on saprobic soil fungi, some aspects may also be generalizable to other fungal groups. For example, future work should test if hyphal density is also a better predictor for soil aggregation ability than hyphal biomass production in arbuscular mycorrhizal fungi. On the other hand, it will also be important to extend the dataset of fungal traits and soil aggregation beyond soil saprobes, since the relative importance of traits and trait combinations could vary; for example, since arbuscular mycorrhizal fungi have limited enzymatic abilities (Tisserant et al., 2013), this trait would play no role in that particular group. In the end, our study demonstrates the power of employing a trait-based approach to tackle biological mechanisms of soil aggregation; this can now also be extended to organism groups other than fungi.

## Data Availability Statement

All datasets generated for this study are included in the article/Supplementary Material.

## Author Contributions

AL designed and performed the research. WZ and MR contributed the analytical tools. AL, WZ, KS, RR, and SM provided the experimental data. JR created the phylogenetic tree. AL and MCR wrote the manuscript. All authors contributed to the final version of the manuscript.

## Conflict of Interest

The authors declare that the research was conducted in the absence of any commercial or financial relationships that could be construed as a potential conflict of interest.

## Footnotes

1 https://github.com/cran/quantreg
