# Supplementary material for: Fungal Traits Important for Soil Aggregation
Source: Front Microbiol. 2020 Jan 9;10:2904. doi: 10.3389/fmicb.2019.02904 (PMC6962133; doi:10.3389/fmicb.2019.02904)
Supplement: Supplementary file 1 [file Data_Sheet_1.docx]

Supplementary information

**Title: Fungal traits important for soil aggregation**

(How to be an effective aggregator)

Anika Lehmann ^a, b^, Weishuang Zheng ^c^, Masahiro Ryo ^a, b^, Katharina Soutschek ^a^, Julien Roy ^a, b^, Rebecca Rongstock ^a^, Stefanie Maaß^b, d^, Matthias C. Rillig ^a, b^

^a^ Freie Universität Berlin, Institut für Biologie, Plant Ecology, Altensteinstr. 6, D- 14195 Berlin, Germany

^b^ Berlin-Brandenburg Institute of Advanced Biodiversity Research, D-14195 Berlin, Germany

^c^ PKU-HKUST ShenZhen-Hong Kong Institution, Shenzhen 518057, China

^d^ Universität Potsdam, Institut für Bioologie und Biochemie, Plant Ecology and Nature Conservation, Am Mühlenberg 3, 14476 Potsdam

*Author to whom correspondence should be sent*

Anika Lehmann, Freie Universität Berlin, Institut für Biologie, Altensteinstr. 6, D-14195 Berlin, Germany; Tel +49 30 838 53145; lehmann.anika@googlemail.com

Contents

[Soil aggregation study collection focusing on filamentous fungi 2](#_Toc518989708)

[Information on the set of 31 fungal strains 3](#_Toc518989709)

[Protocols for experimental setups and measurements 4](#_Toc518989710)

[Overall effect of saprobic fungi on soil aggregate formation capacity 6](#_Toc518989711)

[Test for principal component axes significance 7](#_Toc518989712)

[Test for localization of phyla with respect to principal component axes 8](#_Toc518989713)

[Test for collinearity among traits 9](#_Toc518989714)

[Test for phylogenetic signal 10](#_Toc518989715)

[References 12](#_Toc518989716)

## Soil aggregation study collection focusing on filamentous fungi

**Table S1**. Overview of studies investigating fungal mediated soil aggregation ability. Study collection is derived from Lehmann et al.(Lehmann et al., 2017). Publications are grouped into studies examining both soil aggregation ability and fungal traits (“SA & Traits”) or only soil aggregation ability (“SA only”). The fungal group (arbuscular mycorrhizal fungi, ectomycorrhizal fungi, saprobic fungi), number of fungal strains or mutants (> 3), number of traits measured for these fungal strains or mutants and the type of trait investigated are presented.

| study | study focus | fungal group | no. of fungal strains/ mutants | no. traits measured | traits measured |
| --- | --- | --- | --- | --- | --- |
| (Alguacil et al., 2008) | SA & Traits | AMF | 3 | 2 | enzymes in soil, root colonization |
| (Alguacil et al., 2004) | SA & Traits | AMF | 3 | 2 | enzymes in soil, root colonization |
| Caravaca (Caravaca et al., 2004) | SA & Traits | AMF & SF | 3 | 2 | enzymes in soil, root colonization |
| (Caravaca et al., 2006) | SA & Traits | AMF | 3 | 2 | enzymes in soil, root colonization |
| (Lynch and Elliott, 1983) | SA & Traits | SF | 3 | 1 | Biomass |
| (Martens and Frankenberger, 1992) | SA & Traits | SF | 3 | 1 | exo-biopolymers |
| (Rillig et al., 2005) | SA & Traits | AMF | 6 | 2 | biomass, hyphal length |
| (Schreiner and Bethlenfalvay, 1997) | SA & Traits | AMF | 3 | 2 | root colonization, hyphal length |
| (Schreiner et al., 1997) | SA & Traits | AMF | 3 | 2 | root colonization, no. of spores |
| (Tisdall et al., 2012) | SA & Traits | SF | 6 | 3 | hyphal density, (abrasion resistance, tensile strength) |
| (Wu et al., 2008) | SA & Traits | AMF | 3 | 3 | enzymes in soil, root colonization, hyphal length |
| (Zheng et al., 2014) | SA & Traits | ECM | 9 | 2 | hyphal length, mycelium hydrophobicity |
| (Chapman and Lynch, 1985) | SA only | SF | 5 | 0 | none |
| (Daynes et al., 2012) | SA only | SF | 85 | 0 | none |
| (Gilmour et al., 1949) | SA only | SF | 8 | 0 | none |
| (Griffiths and Jones, 1965) | SA only | SF | 4 | 0 | none |
| (Martin and Anderson, 1943) | SA only | SF | 5 | 0 | none |
| (Martin et al., 1958) | SA only | SF | 30 | 0 | none |
| (Martin and Richards, 1963) | SA only | SF | 7 | 0 | none |
| (McCalla, 1950) | SA only | SF | 3 | 0 | none |

## Information on the set of 31 fungal strains

**Table S2** Information about phylum, order, taxon name and Deutsche Sammlung von Mikroorganismen und Zellkulturen (German Collection of Microorganisms and Cell Cultures GmbH, DSMZ) accession numbers of the 31 fungal strains used in this study.

| **strain ID** | **DSMZ accession number** | **Phylum** | **Order** | **Taxon identification *** |
| --- | --- | --- | --- | --- |
| RLCS10 | DSM100286 | Ascomycota | Pleosporales | Alternaria alternata |
| RLCS21 | DSM100327 | Ascomycota | Pleosporales | Pyrenochaetopsis leptospora |
| RLCS22 | DSM100401 | Ascomycota | Pleosporales | Paraphoma chrysanthemicola |
| RLCS12 | DSM100405 | Ascomycota | Pleosporales | Didymellaceae strain 1 |
| RLCS14 | DSM100404 | Ascomycota | Pleosporales | Didymellaceae strain 2 |
| RLCS30 | DSM100291 | Ascomycota | Chaetothyriales | Exophiala equina |
| RLCS31 | DSM100328 | Ascomycota | Chaetothyriales | Cyphellophora sp. |
| RLCS28 | DSM100323 | Ascomycota | Helotiales | Tricladium sp. |
| RLCS26 | DSM100330 | Ascomycota | Helotiales | Tetracladium marchalianum |
| RLCS25 | DSM100292 | Ascomycota | Hypocreales | Hydropisphaera sp. |
| RLCS20 | DSM100329 | Ascomycota | Hypocreales | Purpureocillium lilacinum |
| RLCS24 | DSM100410 | Ascomycota | Hypocreales | Metarhizium marquandii |
| RLCS23 | DSM101519 | Ascomycota | Hypocreales | Stachybotryaceae strain 1 |
| RLCS05 | DSM100403 | Ascomycota | Hypocreales | Fusarium sp. |
| RLCS08 | DSM100325 | Ascomycota | Hypocreales | Gibberella tricincta |
| RLCS18 | DSM100287 | Ascomycota | Hypocreales | Gibberella sp. |
| RLCS13 | DSM100290 | Ascomycota | Hypocreales | Fusarium solani |
| RLCS27 | DSM100326 | Ascomycota | Sordariales | Thielavia inaequalis |
| RLCS06 | DSM100400 | Ascomycota | Sordariales | Chaetomium angustispirale |
| RLCS07 | DSM100284 | Ascomycota | Xylariales | Amphisphaeriaceae strain 1 |
| RLCS29 | DSM100288 | Basidiomycota | Agaricales | Macrolepiota excoriata |
| RLCS17 | DSM100324 | Basidiomycota | Agaricales | Clitopilus sp. |
| RLCS16 | DSM100408 | Basidiomycota | Agaricales | Pleurotus pulmonarius |
| RLCS09 | DSM100406 | Basidiomycota | Polyporales | Trametes versicolor |
| RLCS03 | DSM100285 | Mucoromycota | Mortierellales | Mortierella alpina strain 1 |
| RLCS11 | DSM100289 | Mucoromycota | Mortierellales | Mortierella alpina strain 2 |
| RLCS15 | DSM100402 | Mucoromycota | Mortierellales | Mortierella elongata strain 1 |
| RLCS02 | DSM100407 | Mucoromycota | Mortierellales | Mortierella elongata strain 2 |
| RLCS04 | DSM100322 | Mucoromycota | Mortierellales | Mortierella exigua |
| RLCS01 | DSM100293 | Mucoromycota | Mucorales | Mucor fragilis |
| RLCS19 | DSM100331 | Mucoromycota | Umbelopsidales | Umbelopsis isabellina |

*best resolved tree annotation passing 80% threshold of bootstrap approach

## Protocols for experimental setups and measurements

We include here previously measured trait data from the articles Zheng et al. (Zheng et al., 2018) and Lehmann et al. (Lehmann et al., 2018). For the sake of completeness, we present here the corresponding protocols:

**Experiment: measuring hyphal branching angle, internodal length and diameter.** The measurement of the hyphal branching angle (BA), internodal length (IL) and diameter (D) occurred on single concavity slides (Lehmann et al (in press)). Since some of the 31 fungal strains had very densely growing colonies, we reduced the PDA concentration to 10% while adding agar to reach 15 g/L concentration and hence create solid but relative nutrient-reduced agar (for all species). This way, we were able to identify single hyphae for all fungal strains. The medium was applied as a 150 µl droplet and flattened by a cover slide until the medium solidified. Finally, a pre-sterilized poppy seed carrying the target fungal strain was placed in the center of the concavity. Subsequently, individual slides were put in a 9 cm Petri dish containing a 5 mm layer of agar to maintain high humidity; for each strain five slides were prepared. The plates were sealed and stored at room temperature (22°C) in the dark. When the fungal mycelium covered half of the concavity area, we started the examination under the microscope (Leica DM2500, brightfield, 200x). For this, we investigated the mycelium from the rim inwards towards the poppy seed. Five hyphae were chosen randomly to measure the first branching angle, the internodal lengths between first and second branch and the hyphal diameters within the first internodal segment via Leica Application Suite (LAS Version 4.8.0). Trait values for the subsamples were merged to one mean value per replicate.

**Experiment: measuring mycelium complexity, lacunarity and hyphal surface area.** In order to measure the mycelial complexity and heterogeneity (Lehmann et al. (Lehmann et al., 2018)), we made use of the same approach as applied in experiment 1 but produced eight replicates per fungal strain. At harvest, we focused on the outer 200 µm of the mycelium under the microscope (Leica DM2500, brightfield, 200x). The goal was to generate high contrast grayscale photos (Leica DFC290) with ideally white background and black hyphae. Three photos were obtained from each slide. The field of view was chosen randomly from the colony edge. Furthermore, photos needed to be processed prior to image analysis. Therefore, we converted the photos to 8-bit images in ImageJ and skeletonized the hyphae by a thinning algorithm (Zhang and Suen, 1984). Subsequently, images were transferred to Adobe Illustrator (CS6, v.16.0.0) to reconnect skeletonized hyphae, remove image artifacts and adjust the line thickness to the mean hyphal diameter obtained in experiment 1. As shown recently (Lehmann et al (in press)) data derived from skeletonized and adjusted diameter images are strongly correlating hence no bias is implemented. Finally, the fractal dimensions of the processed images were analyzed with the ImageJ plug-in FracLac (Karperien, 1999-2013). Box counting dimension (Db) and lacunarity were chosen as metrics for mycelium complexity and heterogeneity, respectively. For the measurements, we applied default settings with rotational orientations in analyses. The subsample data were merged to one trait value per replicate.

Using the processed images for fractal dimension analysis, we obtained hyphal length data by WinRHIZO Pro (v.2007d, Regent Instrument Inc., Quebec, Canada). Assuming hyphae as cylindrical constructs, we combined hyphal length with hyphal diameter data to estimate hyphal surface area (HSA in µm²) of the 31 fungal strains by using the equation: HAS= 2 π r h + 2 π r, where r equals the ½ of the hyphal diameter (D) and h represents the hyphal length. Again, subsample data were merged to one trait value per replicate.

**Experiment: measuring laccase, cellobiohydrolase, acid phosphatase and leucine aminopeptidase activity.** The enzymatic activities were measured on fungal tissue derived from the same approach, as applied for measuring fungal biomass density, with six replicates per fungal strain. Thus we were able to test medium free tissue. Each individual colony was split in two halves which were either used for HPB or enzymatic activity test. For measuring enzymatic activity (U *mg_(dw)_^-1^) of laccase (Lac; lignin degradation), cellobiohydrolase (Cel; cellulose degradation), acid phosphatase (Pho; releases free attached phosphoryl groups) and leucine aminopeptidase (Leu; hydrolysis of peptides) eight pieces of fungal tissue (3-5 mm²) from the peripheral zone of each individual colony were extracted and tested by a modified microplate photometric method by Courtey et al. (2006). Briefly, fungal tissue was incubated with the corresponding test substrate at 37° for 15 min, followed by measuring absorbance at 410 nm in a microplate reader (Bio-RAD, USA). We defined one unit (U) of enzymatic activity as 1 µl of released substrate per min. Finally, we standardized enzymatic activity by mycelial dry weight. Again, subsample data were merged to one trait value per replicate.

## Overall effect of saprobic fungi on soil aggregate formation capacity


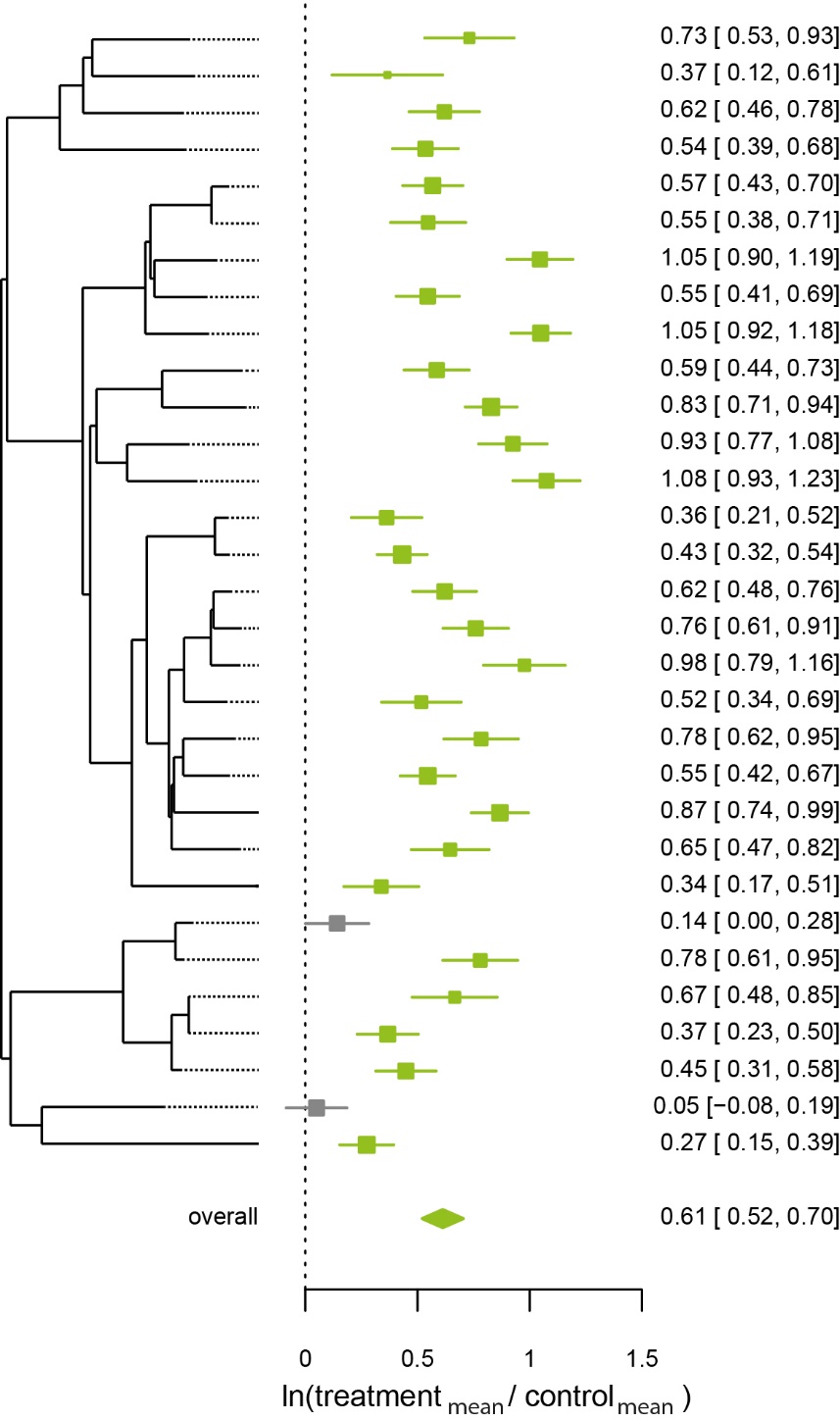


**Fig. S1** Overall effect of fungal mediated soil aggregate formation capacity. Natural logarithm response ratios of the fungal treatment means and control means were incorporated in a random effects model (function rma.uni() in package “metafor” (Viechtbauer, 2010)) with implemented common control correction (function commonControl() in package “metagear” (Lajeunesse, 2016)) to estimate the overall effect (diamond shape at the figure bottom). Green symbols denote positive (confidence intervals are not overlapping the dashed zero line) and grey symbols neutral effects.

## Test for phylogenetic signal

**Table S3.** Phylogenetic signal tested for 15 trait variables and the response variable SAF using the phylogenetic autocorrelation index Moran’s I using R package “phylosignal” (Keck et al., 2016).

| Trait variables | Moran's I | p-value |
| --- | --- | --- |
| SAF | -0.0063 | 0.318 |
| **HAS** | **0.305907** | **0.001** |
| **HLs** | **0.167356** | **0.007** |
| **Db** | **0.094909** | **0.03** |
| L | -0.05457 | 0.674 |
| BA | 0.036896 | 0.119 |
| Db | -0.11682 | 0.91 |
| IL | 0.001451 | 0.214 |
| Kr | -0.00428 | 0.319 |
| PT | 0.018418 | 0.075 |
| Den | 0.048347 | 0.098 |
| HPB | -0.0452 | 0.51 |
| Lac | 0.008605 | 0.147 |
| Leu | -0.00428 | 0.23 |
| **Cel** | **0.14399** | **0.01** |
| Pho | -0.0248 | 0.401 |

For four traits a phylogenetic signal was detected. However, these were not among those traits with identified importance for soil aggregate formation capacity (SAF). Hence, phylogenetic correction for relationships depicted in Fig. 2 was not necessary.

## Test for collinearity among traits


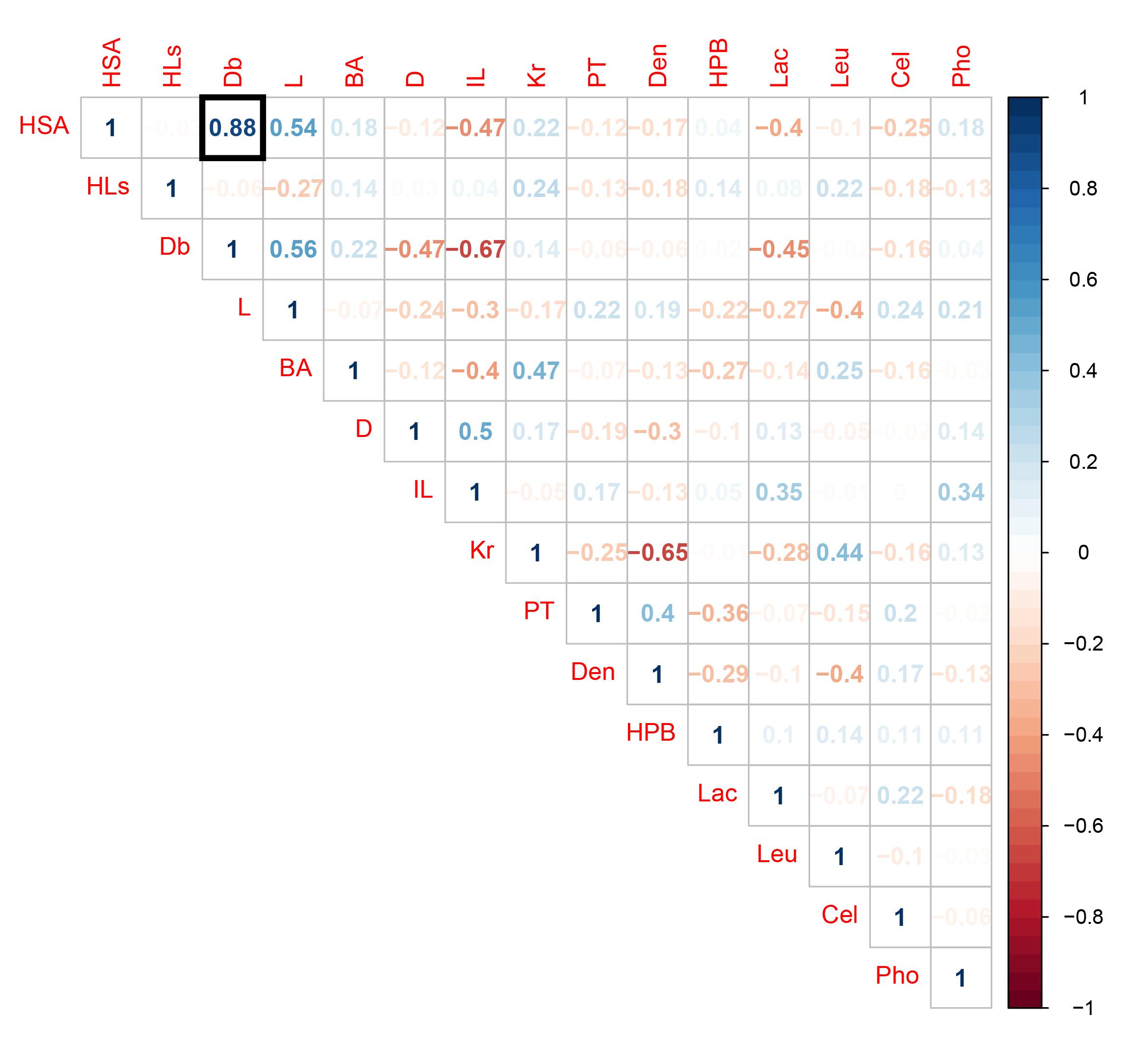


**Fig. S2.** Test for collinearity between 15 trait variables included in the random forest analysis. Collinearity threshold was set at |r|>0.7; r = Pearson’s rho (Bergmann et al., 2017). The black frame marks the correlation coefficient exceeding the threshold. Note: Spearman’s rho yields similar results as Person’s rho and detects solely HSA - Db collinearity.

## Test for localization of phyla with respect to principal component axes


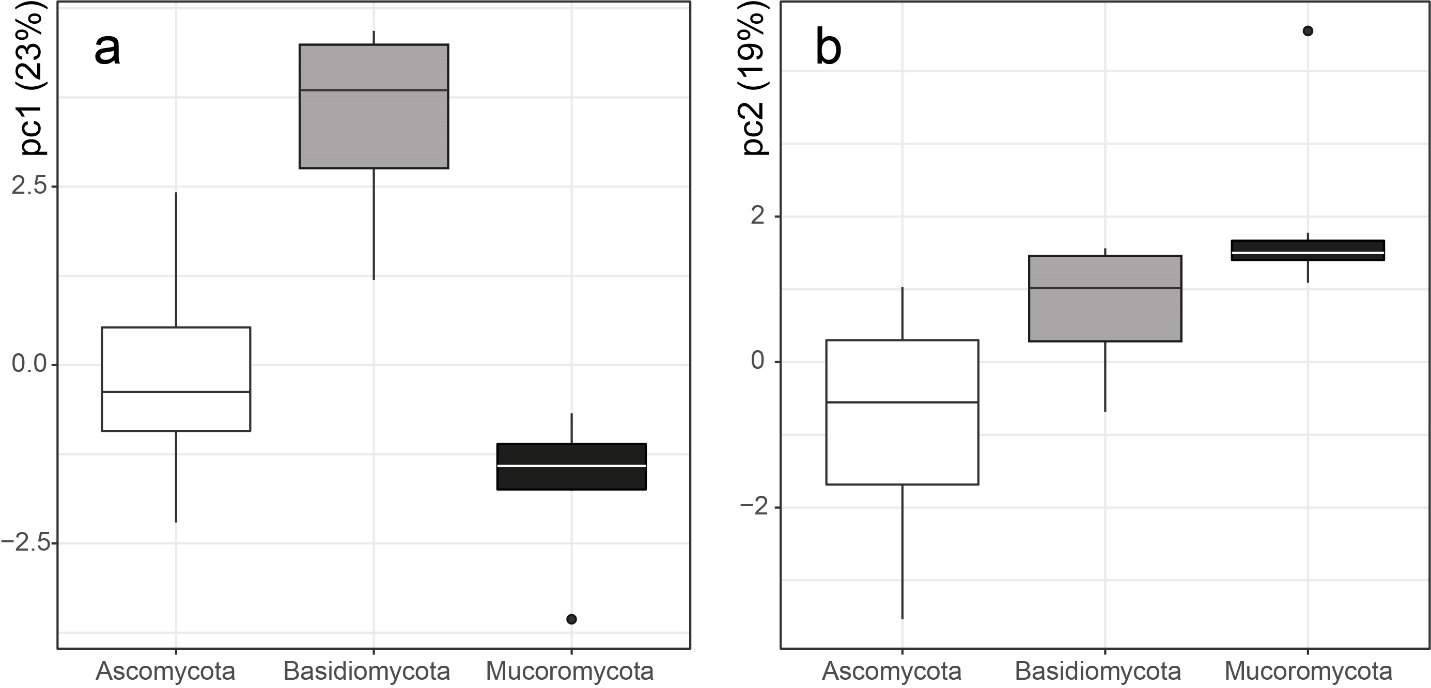


**Fig. S3**. Separation of phyla with respect to PC axis 1 and 2 as the significant representatives of the 15-dimensional trait space. Difference between phyla for PC axes were tested by analysis of variance. With TukeyHSD test pairwise comparisons were tested for significant differences. For PC axis 1 all pairwise comparisons were significantly different from each other (B-A: p<000.1; M-A: p=0.02; M-B: p<0.0001) while for PC axis 2 only Ascomycota and Mucoromycota were significantly different (B-A: p=0.09, M-A: p=0.0001, M-B: p=0.32).

## Test for principal component axes significance

**Table S4.** Significance test for PCA axis loadings using package “ade4” (Dray and Dufour, 2007; Dray et al., 2007; Dray, 2008) with 999 permutations.

| PCA | Obs | Std.Obs | p |
| --- | --- | --- | --- |
| **Axis1** | **0.61122** | **3.012327** | **0.006** |
| **Axis2** | **0.684416** | **4.261694** | **0.001** |
| Axis3 | 0.527843 | -0.45928 | 0.642 |
| Axis4 | 0.54416 | -0.35256 | 0.623 |
| Axis5 | 0.50274 | -1.74368 | 0.983 |
| Axis6 | 0.544195 | -1.20106 | 0.899 |
| Axis7 | 0.566284 | -0.46567 | 0.664 |
| Axis8 | 0.583631 | -1.25759 | 0.904 |
| Axis9 | 0.632945 | 2.009737 | 0.033 |
| Axis10 | 0.699143 | 3.432537 | 0.002 |
| Axis11 | 0.642455 | 1.253162 | 0.12 |
| Axis12 | 0.807911 | 5.126565 | 0.001 |
| Axis13 | 0.704958 | 1.619648 | 0.068 |
| Axis14 | 0.874934 | 4.656843 | 0.001 |
| Axis15 | 0.960331 | 7.554809 | 0.001 |
| Axis16 | 1 | 4.431028 | 0.001 |

**Table S5.** Linear and quantile regression outcomes. Note: For the regression of SAF and Den, SAF needed to be log-transformed to fulfill model assumptions. Data distribution presented in Fig. 2 based on untransformed data.

|  |  | Linear regression | | | Quantile regression | | |
| --- | --- | --- | --- | --- | --- | --- | --- |
| Y | X | equation | R²adj | p | quantile | equation | p |
| log(SAF) | Den | y= 3.50x + 1.62 | 0.23 | 0.004 | 0.05 | y= 17.88x + 3.45 | 0.28 |
|  |  |  |  |  | 0.25 | y= 12.02x + 4.55 | 0.49 |
|  |  |  |  |  | 0.75 | y= 30.88x + 5.56 | 0.01 |
|  |  |  |  |  | 0.95 | y= 26.93x + 7.68 | 0.09 |
|  |  |  |  |  |  |  |  |
| SAF | Leu | y= -0.48x + 7.42 | 0.13 | 0.03 | 0.05 | y= -0.35x - 4.97 | 0.17 |
|  |  |  |  |  | 0.25 | y= -0.26x + 6.27 | 0.43 |
|  |  |  |  |  | 0.75 | y= -0.62x + 8.99 | 0.12 |
|  |  |  |  |  | 0.95 | y= -0.85x + 10.62 | 0.0004 |
|  |  |  |  |  |  |  |  |
| SAF | Phylogeny | y= 0.70x + 6.71 | 0.1 | 0.05 | 0.05 | y= 0.81x + 4.42 | 0.01 |
|  |  |  |  |  | 0.25 | y= 0.83x + 5.45 | 0.05 |
|  |  |  |  |  | 0.75 | y= 0.63x + 7.91 | 0.31 |
|  |  |  |  |  | 0.95 | y= 1.21x + 9.71 | 0.05 |

## References

Alguacil, M.D., Caravaca, F., Diaz, G., Marin, P., Roldan, A., 2004. Establishment of Retama sphaerocarpa L. seedlings on a degraded semiarid soil as influenced by mycorrhizal inoculation and sewage-sludge amendment. Journal of Plant Nutrition and Soil Science 167, 637-644.

Alguacil, M.M., Caravaca, F., Azcon, R., Roldan, A., 2008. Changes in biological activity of a degraded Mediterranean soil after using microbially-treated dry olive cake as a biosolid amendment and arbuscular mycorrhizal fungi. European Journal of Soil Biology 44, 347-354.

Bergmann, J., Ryo, M., Prati, D., Hempel, S., Rillig, M.C., 2017. Root traits are more than analogues of leaf traits: the case for diaspore mass. New Phytologist 216, 1130-1139.

Caravaca, F., Alguacil, M.M., Azcon, R., Diaz, G., Roldan, A., 2004. Comparing the effectiveness of mycorrhizal inoculation and amendment with sugar beet, rock phosphate and Aspergillus niger to enhance field performance of the leguminous shrub Dorycnium pentaphyllum L. Applied Soil Ecology 25, 169-180.

Caravaca, F., Tortosa, G., Carrasco, L., Cegarra, J., Roldan, A., 2006. Interaction between AM fungi and a liquid organic amendment with respect to enhancement of the performance of the leguminous shrub Retama sphaerocarpa. Biology and Fertility of Soils 43, 30-38.

Chapman, S.J., Lynch, J.M., 1985. Polysaccharide synthesis by capsular microorganisms in coculture with cellulolytic fungi on straw and stabilization of soil aggregates. Biology and Fertility of Soils 1, 161-166.

Daynes, C.N., Zhang, N., Saleeba, J.A., McGee, P.A., 2012. Soil aggregates formed in vitro by saprotrophic Trichocomaceae have transient water-stability. Soil Biology & Biochemistry 48, 151-161.

Dray, S., 2008. On the number of principal components: A test of dimensionality based on measurements of similarity between matrices. Computational Statistics & Data Analysis 52, 2228-2237.

Dray, S., Dufour, A.B., 2007. The ade4 package: Implementing the duality diagram for ecologists. Journal of Statistical Software 22, 1-20.

Dray, S., Dufour, A.B., Chessel, D., 2007. The ade4 package-II: Two-table and K-table methods. R News 7, 47-52.

Gilmour, C.M., Allen, O.N., Truog, E., 1949. Soil Aggregation as Influenced by the Growth of Mold Species, Kind of Soil, and Organic Matter. Soil Science Society of America Journal 13, 292-296.

Griffiths, E., Jones, D., 1965. Microbiological aspects of soil structure. I. Relationships between organic amendments, microbial colonization, and changes in aggregate stability. . Plant and Soil 23, 17-33.

Karperien, A., 1999-2013. FracLac for ImageJ.

Keck, F., Rimet, F., Bouchez, A., Franc, A., 2016. phylosignal: an R package to measure, test, and explore the phylogenetic signal. Ecology and Evolution 6, 2774-2780.

Lajeunesse, M.J., 2016. Facilitating systematic reviews, data extraction and meta-analysis with the metagear package for r. Methods in Ecology and Evolution 7, 323-330.

Lehmann, A., Zheng, W., Soutschek, K., Rillig, M.C., 2018. How to build a mycelium: tradeoffs in fungal architectural traits. bioRxiv.

Lehmann, A., Zheng, W.S., Rillig, M.C., 2017. Soil biota contributions to soil aggregation. Nature Ecology & Evolution 1, 1828-+.

Lynch, J.M., Elliott, L.F., 1983. Aggregate stabilization of volcanic ash and soil during microbial-degradation of straw. Applied and Environmental Microbiology 45, 1398-1401.

Martens, D.A., Frankenberger, W.T., 1992. Decomposition of bacterial polymers in soil and their influence on soil structure. Biology and Fertility of Soils 13, 65-73.

Martin, J.P., Ervin, J.O., Shepherd, R.A., 1958. Decomposition and aggregating effect of fungus cell material in soil. Soil Science Society of America Journal.

Martin, J.P., Richards, S.J., 1963. Decomposition and binding action of a polysaccharide from *Chromobacterium violaceum* in soil. Journal of Bacteriology 85, 1288-&.

Martin, T.L., Anderson, D.A., 1943. Organic matter decomposition, mold flora, and soil aggregation relationships. Soil Science Society of America Journal 7, 215-217.

McCalla, T.M., 1950. Microorganisms and Soil Structure. Transactions of the Kansas Academy of Science 53, 91-100.

Rillig, M.C., Lutgen, E.R., Ramsey, P.W., Klironomos, J.N., Gannon, J.E., 2005. Microbiota accompanying different arbuscular mycorrhizal fungal isolates influence soil aggregation. Pedobiologia 49, 251-259.

Schreiner, R.P., Bethlenfalvay, G.J., 1997. Plant and soil response to single and mixed species of arbuscular mycorrhizal fungi under fungicide stress. Applied Soil Ecology 7, 93-102.

Schreiner, R.P., Mihara, K.L., McDaniel, H., Bethlenfalvay, G.J., 1997. Mycorrhizal fungi influence plant and soil functions and interactions. Plant and Soil 188, 199-209.

Tisdall, J.M., Nelson, S.E., Wilkinson, K.G., Smith, S.E., McKenzie, B.M., 2012. Stabilisation of soil against wind erosion by six saprotrophic fungi. Soil Biology & Biochemistry 50, 134-141.

Viechtbauer, W., 2010. Conducting Meta-Analyses in R with the metafor Package. Journal of Statistical Software 36, 1-48.

Wu, Q.S., Xia, R.X., Zou, Y.N., 2008. Improved soil structure and citrus growth after inoculation with three arbuscular mycorrhizal fungi under drought stress. European Journal of Soil Biology 44, 122-128.

Zhang, T.Y., Suen, C.Y., 1984. A fast parallel algorithm for thinning digital patterns. Communications of the Acm 27, 236-239.

Zheng, W., Lehmann, A., Ryo, M., Valyi, K., Rillig, M.C., 2018. Growth rate trades off with enzymatic investment in soil filamentous fungi. bioRxiv.

Zheng, W.S., Morris, E.K., Rillig, M.C., 2014. Ectomycorrhizal fungi in association with Pinus sylvestris seedlings promote soil aggregation and soil water repellency. Soil Biology & Biochemistry 78, 326-331.
